# Proposed actions are no actions: re-modeling an ontology design pattern with a realist top-level ontology

**DOI:** 10.1186/2041-1480-3-S2-S2

**Published:** 2012-09-21

**Authors:** Djamila Seddig-Raufie, Ludger Jansen, Daniel Schober, Martin Boeker, Niels Grewe, Stefan Schulz

**Affiliations:** 1 Institute of Medical Biometry and Medical Informatics, Freiburg University Medical Center Freiburg, Germany; 2 Institute of Philosophy, University of Rostock, Germany; 3 Philosophical Institute, RWTH Aachen University, Germany; 4 Institute for Medical Informatics, Statistics and Documentation, Medical University of Graz, Austria

## Abstract

**Background:**

Ontology Design Patterns (ODPs) are representational artifacts devised to offer solutions for recurring ontology design problems. They promise to enhance the ontology building process in terms of flexibility, re-usability and expansion, and to make the result of ontology engineering more predictable. In this paper, we analyze ODP repositories and investigate their relation with upper-level ontologies. In particular, we compare the BioTop upper ontology to the *Action *ODP from the NeOn an ODP repository. In view of the differences in the respective approaches, we investigate whether the *Action *ODP can be embedded into BioTop. We demonstrate that this requires re-interpreting the meaning of classes of the NeOn *Action *ODP in the light of the precepts of realist ontologies.

**Results:**

As a result, the re-design required clarifying the ontological commitment of the ODP classes by assigning them to top-level categories. Thus, ambiguous definitions are avoided. Classes of real entities are clearly distinguished from classes of information artifacts. The proposed approach avoids the commitment to the existence of unclear future entities which underlies the NeOn *Action *ODP. Our re-design is parsimonious in the sense that existing BioTop content proved to be largely sufficient to define the different types of actions and plans.

**Conclusions:**

The proposed model demonstrates that an expressive upper-level ontology provides enough resources and expressivity to represent even complex ODPs, here shown with the different flavors of *Action *as proposed in the NeOn ODP. The advantage of ODP inclusion into a top-level ontology is the given predetermined dependency of each class, an existing backbone structure and well-defined relations. Our comparison shows that the use of some ODPs is more likely to cause problems for ontology developers, rather than to guide them. Besides the structural properties, the explanation of classification results were particularly hard to grasp for 'self-sufficient' ODPs as compared with implemented and 'embedded' upper-level structures which, for example in the case of BioTop, offer a detailed description of classes and relations in an axiomatic network. This ensures unambiguous interpretation and provides more concise constraints to leverage on in the ontology engineering process.

## Background

In this paper we want to show the re-modeling of a specific ODP. We checked whether this ODP can be embedded in BioTop ontology. The results can be downloaded from this link [1]. But what are ODPs? And which repositories exist?

Design patterns are popular in software engineering [2]. Recently, they have also been proposed for ontology building. ODPs claim to be re-usable and standardized solutions to commonly occurring design problems, thus supporting ontology engineers in the efficient development of ontologies. Another advantage is that the resulting artifacts are more manageable, as their design principles are explicitly known. We have investigated three main sources of ODPs:

1. The ***Semantic Web Best Practices and Deployment Working Group ***[3]: The aim of this group is to guide Semantic Web developers to build reusable OWL ontologies. There are some design patterns that can be considered as formalized design patterns, for example, n-ary relations, value partitions, value sets and simple part-whole relations in OWL Ontologies. These are patterns which are well documented with block diagrams. The other ODPs from this source are non-formalized patterns that are explanations or documentations of how to design a pattern rather than design patterns in a strict sense.

2. The ***Ontology Design Patterns (ODPs) Public Catalog ***focuses on the domain of biological knowledge [4]. It contains OWL files describing an ODP that can be downloaded from the Sourceforge project site [5]. The Ontology Design Patterns (ODPs) Public Catalog distinguishes between Extensional ODPs, Good Practice ODPs and Modeling ODPs [6,7]. Extensional ODPs are helpful by expanding the limitations faced by OWL. While Good Practice ODPs aim at obtaining ontologies which comply with predefined quality criteria, Modeling ODPs assist in modeling domains according to their concrete requirements.

3. The resource ***OntologyDesignPatterns.org (ODP) ***[8] has been developed under the auspices of the European NeOn project. The aims of these ODPs, in contrast to Public Catalog ODPs, are to enable the use and development of networked ontologies. A survey of the classification of ODPs used by NeOn [8,11] and the number and definitions [8-10,12] of ontologies assigned to these classes can be found in Table 1.

**Table 1:** NeOn Ontology Design Pattern types

Types of ODPs	Division of ODP Types	Explanation	Number of ODPs
Structural ODPs	Logical ODPs	A Logical OP is a formal expression, whose only parts are expressions from a logical vocabulary, e.g. OWL DL, that solves a problem of expressivity [8].	13
	Architectural ODPs	Logical ODPs or compositions of them that are used exclusively in the design of an ontology. An Architectural ODPs is also a content-independent structure. In other words, an Architectural ODPs is supposed to characterize the overall structure of an ontology. In simple terms, an Architectural ODP dictates "how an ontology should look like" [12].	1

Correspondence ODPs	Re-engineering ODPs	Reengineering ODPs are transformation rules applied in order to create a new ontology (target model) starting from elements of a source model [8].	12
	Alignment ODPs	Alignment ODPs refer to correspondences between ontologies. Each pattern models a relation between two entities or sets of entities in two ontologies. Instantiation of an Alignment ODP results in a correspondence between elements of two given ontologies [8].	13

Presentation ODPs	Naming ODPs	Naming ODPs are conventions on how to create names for namespaces, files, and ontology elements in general (classes, properties, etc.) [8].	Containing no ODPs
	Annotation ODPs	Annotation ODPs provide annotation properties or annotation property schemas that are meant to improve the understandability of ontologies and their elements [8].	Containing no ODPs

Content ODPs (CPs)		Content ODPs are distinguished networked ontologies and have their own namespace. They cover a specific set of competency questions (requirements), which represent the problem they provide a solution for. Furthermore, Content ODPs show certain characteristics, i.e. they are: computational, small, autonomous, hierarchical, cognitively relevant, linguistically relevant, and best practices [8].	92

Reasoning ODPs		Reasoning ODPs applications of Logical OPs oriented to obtain certain reasoning results, based on the behavior implemented in a reasoning engine [8][9]. Examples for Reasoning ODPs are classification, subsumption, inheritance, etc [8].	Containing no ODPs

Lexico-Syntactic ODPs		Lexico-Syntactic ODPs are linguistic structures or schemas that consist of certain types of words following a specific order, and that permit to generalize and extract some conclusions about the meaning they express. They are useful for associating simple Logical and Content ODPs with natural language sentences, e.g. for didactic purposes [10].	20

The NeOn project distinguishes several types of Ontology Design Patterns (ODPs). The table gives an overview about theses ODP classes and the number of ODPs assigned to them in the NeOn repository; February 2010.

Despite the wealth of available ODPs, at least the content of the NeOn repositories appears to be rather idiosyncratic, mainly due to the fact that their ODPs refrain from a clear ontological commitment and leave the final interpretation to the user.

In particular, we investigated the following:

1. Which elements (classes and relations) of ODPs are already expressed by BioTop axioms?

2. To what extent and how can existing ODPs be re-interpreted or adapted for inclusion into top-level ontologies like, e.g., BioTop?

3. Which parts of ODPs can be re-designed as extensions to BioTop?

We identified corresponding representations in BioTop by comparing the intended meaning of ODP and BioTop representations by structural and logical analysis.

## Methods

### BioTop

We address this problem by proposing an ontology engineering approach rooted in a philosophically founded ontological top-level, using the BioTop ontology [13], a publically available upper-domain level for the life sciences [14,15]. BioTop provides foundational classes and relations embedded in richly axiomatized definitions. Its set of relations is considered to be exhaustive so that ontology developers only need to subclass existing classes and define them by adding restrictions using the existing relations (OWL object properties). The addition of new relations for domain ontologies that build on BioTop is discouraged. BioTop is compatible with the major top-level ontologies like BFO [16], DOLCE [17], and the OBO Relation Ontology [18].

### The *Action *ODP

**Figure 1:**
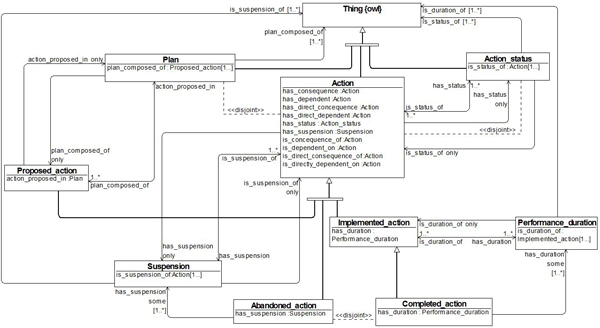
**The Action ODP from the NeOn Project**. The Action ODP from the NeOn Project models actions as being performed, abandoned, proposed or planned. Every action has status and duration in time. The diagram shows an action class with his subclasses. The subclasses of action class represent different types of actions.

As an example for our investigation, we choose the *Action *ODP from the NeOn repository [19], because it addresses a central modeling challenge and is a good comparison to detect the differences between *Action *ODP and BioTop top-level ontology. The *Action *ODP, depicted in Figure 1, is classified by NeOn as a Content ODP. It aims at representing actions as being proposed, planned, performed or abandoned, together with their status and duration [19]. For this purpose it includes action properties such as 'status' and 'duration'. The class *Action *is described as: "The process of doing something. An action is performed by an agent." [19] A link to a class called *Action_status *is used to differentiate actions in terms of being proposed, implemented (and possibly completed), or abandoned. As a result, *Proposed_action*, *Abandoned_action*, *Completed_action*, and *Implemented_action *are defined. Together with *Plan*, *Action_status*, *Suspension*, and *Performance_duration*, they can, furthermore, be related by means of ten relations like **has_consequence**, **has_dependent **etc. The class *Performance_duration *indicates the time interval in which an action is performed. Finally, *Plan *is introduced as a "set of proposed actions and the sequence in which to perform them" [19].

### Methodological assumptions

To show that ODPs can be embedded in top-level ontologies, our immediate goal was to make the *Action *ODP compatible with BioTop. In order to do so, we followed the realist approach to ontology design that inspired the development of BioTop. The realist approach assumes that every class must be justified by individuals in the real world which is to a large extent ontologically independent of us, i.e. it would also exist without any human observers. The principle of ontological realism "is based on the idea that the most effective way to ensure mutual consistency of ontologies over time and to ensure that ontologies are maintained in such a way as to keep pace with advances in empirical research is to view ontologies as representations of the reality that is described by science" [20].

For our case study, this implies that the ODP classes must comply with BioTop's rigid upper-level categories, especially regarding the distinction between real objects and information object. An information object is a piece of information that exists independently of any particular material carrier [13].

In addition, we followed the economic maxim that the number of new representational units should be minimal. Most importantly, no new relations (i.e. object properties in Protégé OWL lingo) should be added, as BioTop already contains a well-defined set of formal relations.

## Results

We were able completely to re-model the *Action *ODP from the NeOn repository with equivalent structures in BioTop. In doing so, however, several shortcomings of the *Action *ODP were uncovered and resolved. BioTop already contains a class *Action *which is a subclass of *Process*. Actions are defined in BioTop as processes promoted by an agent, having a clear role distinction between agent and other non-agentive process participants. By definition, processes have temporal parts, i.e. there is no time in which all parts of a process are simultaneously present. Processes have physical or abstract entities as participants:

*Process *subclassOf **hasParticipant **some *Particular*

The object property **hasAgent **is a subrelation of **hasParticipant**.

*Action *equivalentTo *Process *and **hasAgent **some *Particular*

Additionally,

*Action *subClassOf **hasDuration **some *TimeInterval*

In the *Action *ODP, the class *Action *can be refined by a number of specific modifiers like "suspended", "completed" or "planned". Although BioTop does not contain these modifiers, this does not preclude more detailed classifications of subclassifying actions in terms of suspension or completion. The axioms in BioTop that characterize the class *Action *tell us that actions, in order to exist, must have an agent and a duration. Proposed actions (in the sense of the ODP), in contrast, have no duration, and the proposed agent does not necessarily exist at the time of the proposal. As a consequence of BioTop's realist view of the world, we cannot assert the existence of entities and relations which are supposed to exist in the future only. Therefore what is called *Proposed_action *in the ODP is not an *Action *in BioTop.

Also, the BioTop axiom

*Action ***hasAgent **some *Particular*

posits an ontological dependence of actions on existing agents: if there is no agent, there cannot be an action.

Like the *Action *ODP, BioTop already includes the class *Plan *with the axiom:

*Plan *subClassOf *InformationObject *and **hasRealization **only *Process*

If aligned with BFO, *InformationObject *would be classified as *GenericallyDependentContinuant*. It is disjoint from *RealizableEntity*, which has only *Disposition*, *Function *and *Role *as subclasses [21]. In contrast, BioTop subscribes to a broader notion of realization, which also includes the relation between an *InformationObject *and a related *Process *[14].

Accordingly, we can define a plan for a specific action *X *as:

*X_Plan *equivalentTo *Plan *and **hasRealization **only *X*

A plan for *X *is therefore only realized when an action of type *X *is accomplished. This is not the case if *X *is merely proposed. The class *X_proposed *is not a subclass of *X*, because it has no (real) duration and agent. Proposed actions are no more actions than fake money is money, than a prevented victory is a victory or than pretending doctors are doctors. A proposed action is rather the content of a proposal, where a proposal could be an information object or some action like a speech act that formulates the plan. A plan can be anything outlining the course of an action, from a documentation of medical procedures and the schedule of operations in a hospital to mere mental entities like the intention to have lunch at noon. Likewise, a suspended action *X *is not an action of type *X *because defining characteristics of *X *may be missing.

In order to illustrate our discussion, think of a surgical action like an *Endoscopic Removal of Foreign Body from Stomach *(e.g. in a child who swallowed a marble). For the sake of brevity, we will refer to this procedure type as *X*. (Thus in what follows, '*X*' is a constant. At the same time, many of the following formulae can be used as schematic guides how to deal with the modifiers in question generally.) In a simplified form we can describe *X *as follows: every instance of *X *begins with an endoscopy preparation (*a*), followed by the introduction of the endoscope (*b*), endoscopic exploration (*c*), grasping of the foreign body (*d*), and extraction of the endoscope with the foreign body (*e*). A description of this is outlined in the information object *X_Plan*. This plan is realized only by actions that correspond to the sequence *abcde*. If any of these sub-actions is missing, the action is no longer of the type *X*. *X_Plan *is therefore only realized when *X *is fully accomplished. As *X *has necessarily all temporal parts *a*-*e*, *X_implemented *is not a subclass of *X*, because it may still be in the phase *a *or *b*, lacking the remaining sequential processes *c-e*. The same applies to *X_abandoned *(e.g., the action is incomplete because no foreign body was found, i.e. no *d *is performed). Rooted in realist philosophy, and in accordance with common sense, BioTop makes a clear distinction between information objects like plans and real processes. It is therefore not compatible with the *Action *ODP as depicted in Figure 1, which obfuscates the ontological distinction between real and hypothetic entities. In what follows, we set out alternative models to account for the different 'flavors' of actions contained in the *Action *ODP in a way compatible with both realism and common sense.

### Proposed actions

A proposed action is not an action. It is a refined plan and hence resides in a completely different top-level category, i.e. the category *InformationObject*. Thus, a proposed action is a proposal of an action - and not an action that has been proposed. This implies that the difference between *Proposed_action *and *Action *is not merely epistemic. Many instances of the class *Proposed*_*action *(i.e. many action proposals) are never realized, thus having no counterpart among the instances of the class *Action*.

Plans for actions of type *X *can be refined by adding further restrictions to the realization class of the plan. E.g., the general plan for *Endoscopic Removal of Foreign Body from Stomach *is refined in terms of a patient, a doctor, an operation room, a time slot etc. according to the following pattern:

*Specified_X_Plan_ByDoctorInHospital *equivalentTo

*Plan *and **hasRealization **only (*X *and **hasAgent **some *Doctor*

and **hasLocus **some *Hospital*)

It should be noted that a *Specified_X_Plan *as such does not have an agent. It is the realization of the plan that has agents, locations etc.

The class *SpecifiedPlan *can be fully defined within BioTop:

*Specified_X_Plan *equivalentTo

**outcomeOf **some *PlanSpecificationAction *and

**hasParticipant **some *X_Plan*

The advantage over the NeOn ODP is that *PlanSpecificationAction*, as a separate action, may have a different agent: The person who schedules a certain surgical intervention is not necessarily identical with the physician who performs it, and both may be different from the person that has formulated the generic plan [22] of which this operation is a realization.

### Implemented action

Here, the action is ongoing, and it may lack some of the features that make it an instance of the type *X_completed*. For instance, the stomach is being explored, but the foreign body not yet found. In such a case, only the initial sequential parts of the original plan have been executed. As a plan is only fully realized at the end of the action, an ongoing action realizes a proper part of the plan. E.g. if the whole plan projects the action parts *a*, *b*, *c*, *d*, *e*, an action which is ongoing in stage *c *has only realized the sub-plans *a *and *b*. Therefore:

*X_implemented *equivalentTo

*Action *and **realizationOf **some

((**abstractPartOf **some *X_Plan*) or *X_Plan*)

(The fact that, e.g. sub-plan *c *cannot be executed without being preceded by *a *and *b *requires additional axioms).

### Completed action

An action is completed if and only if the plan has been fully executed:

*X_completed *equivalentTo

*Action *and **realizationOf **some *X_Plan*

It should be noted that some actions may be completed as soon as they are implemented. This is the case if their *X_Plan *has only one abstract part, like, for example, looking at the Mona Lisa or sitting on the floor.

### Abandoned action

An action is abandoned only if it is no longer being performed and the plan has been executed only partly. In contrast to the implemented action, it is by definition not completed:

*X_NonCompleted *equivalentTo

*Action *and (not **realizationOf **some *X_Plan*) and

**realizationOf **some (**abstractPartOf **some *X_Plan*)

Furthermore, the NeOn *Action *ODP introduces the status variable *Suspension *for permanently or temporarily suspended actions (see also Grewe et. al in this special issue of JBMS). In BioTop we suggest a similar solution, for the lack of a detailed enough time model. However, in order to be consistent with the ontological principles of BioTop, this property needs to be exactly typed. We name it *Inactive*, a subclass of *Quality*, linked to the action by the relation **hasProcessQuality**.

*X_Abandoned *equivalentTo

*X_NonCompleted *and **hasProcessQuality **some *Inactive*

An instance of *X_Abandoned *can permanently bear this quality; then the action is aborted. I can also lose this quality when the action is resumed; then it becomes an instance of implemented action, again. Thus, "abandoned" here means "interrupted" rather than aborted, and were we to introduce new class labels instead of using the NeOn labels, we would be well advised to use *X_Interrupted and X_Aborted *instead. Note that all action classes distinguished here are non-rigid in the sense of OntoClean [23]: an *Action *token is first implemented and eventually completed. Or it is implemented and then abandoned. It may later be re-implemented and completed.

The restricted expressivity of DL does not allow tracking the identity of individuals across classes. Nevertheless the non-rigidity of these classes is an important guide for human ontology developers.

## Discussion

We have presented the results from the comparison of a NeOn ODP with the BioTop upper-level ontology. The NeOn *Action *ODP and the BioTop class *Action *are intended to describe the same phenomena but show fundamental differences. The *Action *ODP presents an *Action *class and different types of action e.g. *Proposed_action*, *Abandoned_action*, *Implemeted_action*, *Completed_action*. The four other classes, namely, *Plan*, *Action_status*, *Suspension*, and *Performance_duration*, are related by ten relations. In BioTop, the class *Action *already exists as a subclass of *Process*, and the class *Plan *as a subclass of *InformationObject*. In BioTop, *Proposed_action *is not a subclass of *Action*, but a subclass of *Plan*.

Our comparison revealed some advantages for BioTop and some disadvantages for NeOn ODPs. These are in particular:

(a) BioTop does not leave the interpretation of the meaning of its classes and relations to the user;

(b) BioTop avoids ambiguous definitions;

(c) BioTop provides a well-structured upper-level;

(d) BioTop provides an exhaustive set of object properties, which is not expected to be refined or enhanced by domain ontologies.

The *Action *ODP has, according to our analysis, the following shortcomings:

(a) it has a complex structure;

(b) it is ambiguous as there is no distinction between real entities and information artifacts;

(c) it introduces ten new object properties that make the pattern more complex than necessary.

These disadvantages of the NeOn ODP can cause problems for ontology developers, when classes are used without a detailed explanation. Moreover, some of the implications of the pattern are plainly false, like, e.g., treating *Proposed_action *as a subclass of *Action*. A proposed action is not an action, just as a purported expert is not an expert and a mock exam not an exam. A proposed action is, in fact, the content of an action proposal. The proposed action does not exist at the time at which the action is proposed, and in case the proposal is never implemented, the proposed action may never exist. For example, in a family there may be several proposals for this year's joint summer vacations, but at most one of these proposed actions can be realized. Thus it is more appropriate to model proposed actions as plans that are being developed or suggested by persons. A plan is the outcome of mental activity and not itself an action performed by an agent.

Our discussion of this NeOn ODP shows that ODPs from NeOn repositories or other repositories like the Public Catalog may be difficult to integrate in ontologies that subscribe to the realist paradigm. They can, however, be used efficiently if the ODPs are rebuilt to fulfill the requirement of realist ontology.

The lack of interpretations of ODPs can lead to a lack of understanding or to misunderstanding on the side of the ontology developer, as it leaves too much leeway for the interpretation of their intended meaning. To create ontologies more efficiently, well-structured ODPs are required that provide adequate elucidations how they should be interpreted with regard to the domain. A good presentation, a better structure, and sufficient explanations for classes and relations can lead to a successful application of ODPs.

Our paper gives some insights into the considerations that have to be taken into account when implementing action representations. Using practical examples, we demonstrated possible misinterpretations which may occur when implementing the proposed *Action *ODP in an upper-level ontology which distinguishes between representing the real world and representing information about the real world.

We have to consider that an ODP proposes a solution for a particular problem. In the end, how ODPs are used depends on the ontology developer who can decide whether she wants to use it or not. Although it is possible to use top-level ontology instead of ODP, they cannot be replaced by top-level ontologies, as the latter introduce class definitions and constraints, but do not provide any solution for a given problem. As an example, the definition of *Action *in BioTop is not an ODP; it is a set of axioms that allow classify entities as instances of *Action*. We therefore recommend developing ODPs closely together with upper-level ontologies. Apart from that, top-level ontologies are possible solutions for who are not familiar with ODPs. As the problems like those which we diagnosed for the *Action *ODP can also be transferred onto other ODPs, we would recommend ODP users to carry out similar analyses on all ODPs they might want to use for their own applications.

## Conclusions

The proposed model demonstrates that an expressive upper-level ontology like BioTop provides enough resources to represent even complex ODPs, as is shown here with the different flavors of *Action *proposed in the NeOn ODP. We identified the following advantages of our approach:

**1. It is explicit **in terms of ontological commitment, i.e. it does not leave the interpretation of the meaning of its classes and relations to the user.

**2. It is parsimonious **in the sense that existing classes and relations in BioTop have proved to be largely sufficient to define the different types of actions and plans. The only auxiliary classes that had to be created were *Inactive *and *PlanSpecificationAction*. No new relations were necessary, whereas the NeOn approach introduces 10 new object properties rendering the pattern more complex than necessary.

**3. It is ontologically clearer **in the sense that ambiguous definitions are avoided. It makes a good distinction between physical entities, processual entities and information artifacts.

**4. It has a simpler and more intuitive notion of existence**. We only have to posit entities which have existed before or do exist at the moment, whereas the *Action *ODP claims existence for unclear future or hypothetic entities, e.g., in the class *Proposed_action*. According to the basic axioms of BioTop, every instance of *Action *must have some agent. As the NeOn ODP conceives of *Proposed_action *as a subtype of *Action*, any instance of *Proposed_action *must have an agent, too. But as proposed actions may never be implemented, the *Action *ODP seems to be committed to postulate the existence of potential or merely possible entities.

**5. It avoids counterintuitive consequences**. Treating *Proposed_action *as a subtype of *Action *yields, e.g., the consequence that there are actions that are never implemented. In fact, there could be proposals for actions that actually exclude each other. But as everyone knows, you cannot eat your cake and keep it. The *Action *ODP, however, would be committed to postulate the existence of both actions.

**6. It is user-friendly**. BioTop's strict division in disjoint partitions and the specification of domain and range restrictions in the definition of object properties guide the user on the right path when extending the ontology. In order to link actions and plans there is no other option in BioTop than using the relation **realizationOf**; and for mereologically relating information entities there is only **abstractPartOf **in BioTop. Hence, compared to self-standing ODPs, patterns that are embedded in a top-level ontology are more user-friendly, as the user profits from inherited constraints.

## Competing interests

The authors declare that they have no competing interests.

## Authors' contributions

SS conceived the research idea, drafted the manuscript. SS, MB, DSR designed the research. DSR performed the analysis and helped drafting the manuscript. SS, LJ, DS, DSR interpreted the results. SS, DS, LJ, MB, NG edited the manuscript. All authors read and approved the final manuscript.
